# Seasonal prevalence and characteristics of low-dose CT detected lung nodules in a general Dutch population

**DOI:** 10.1038/s41598-021-88328-y

**Published:** 2021-04-28

**Authors:** Harriet L. Lancaster, Marjolein A. Heuvelmans, Gert Jan Pelgrim, Mieneke Rook, Marius G. J. Kok, Ahmed Aown, Geertruida H. de Bock, Maarten van den Berge, Harry J. M. Groen, Rozemarijn Vliegenthart

**Affiliations:** 1 Department of Radiology, University Medical Center Groningen, University of Groningen, Hanzeplein 1, 9713 GZ Groningen, The Netherlands; 2 Department of Epidemiology, University Medical Center Groningen, University of Groningen, Groningen, The Netherlands; 3 Martini Hospital Groningen, Groningen, The Netherlands; 4 Department of Pulmonary Diseases, University Medical Center Groningen, University of Groningen, Groningen, The Netherlands

**Keywords:** Diagnostic markers, Epidemiology

## Abstract

We investigated whether presence and characteristics of lung nodules in the general population using low-dose computed tomography (LDCT) varied by season. Imaging in Lifelines (ImaLife) study participants who underwent chest LDCT-scanning between October 2018 and October 2019 were included in this sub-study. Hay fever season (summer) was defined as 1st April to 30th September and Influenza season (winter) as 1st October to 31st March. All lung nodules with volume of ≥ 30 mm^3^ (approximately 3 mm in diameter) were registered. In total, 2496 lung nodules were found in 1312 (38%) of the 3456 included participants (nodules per participant ranging from 1 to 21, median 1). In summer, 711 (54%) participants had 1 or more lung nodule(s) compared to 601 (46%) participants in winter (*p* = 0.002). Of the spherical, perifissural and left-upper-lobe nodules, relatively more were detected in winter, whereas of the polygonal-, irregular-shaped and centrally-calcified nodules, relatively more were detected in summer. Various seasonal diseases with inflammation as underlying pathophysiology may influence presence and characteristics of lung nodules. Further investigation into underlying pathophysiology using short-term LDCT follow-up could help optimize the management strategy for CT-detected lung nodules in clinical practice.

## Introduction

Computed tomography (CT)-detected lung nodules remain a challenge in clinical practice. This is due to the difficulty in distinguishing between their varying etiologies: infection, primary lung cancer, metastasis, or hamartoma to name a few. Lung nodules are identified on 15 to 30% of all computed tomography (CT) scans, and this number is expected to rise with the impending implementation of lung cancer screening in Europe^1^. Early detection through low-dose computed tomography (LDCT) screening resulted in reduced lung cancer mortality in the US National Lung Screening Trial^2^. Consequently, the United States have recommended that high-risk individuals are screened for lung cancer^3–8^. The 10-year follow-up results from the Dutch-Belgium Randomised Lung Cancer Screening trial (NELSON) recently showed that LDCT screening reduces lung cancer mortality by 24% in men and by 33% in women^9^. A positive recommendation for lung cancer screening in Europe is expected^10^. To manage the anticipated increase in LDCT-detected lung nodules in the clinic, appropriate guidelines for management of both routinely and screen detected lung nodules are required. The aforementioned lung cancer screening trials have added fundamental knowledge on lung nodules to the development of existing guidelines, such as the revised Fleischner Society Guidelines 2017^1,11^. However, as valuable as these trials are, they only provide lung nodule information for high-risk individuals, namely current and former smokers.

Thus far, there is limited knowledge on lung nodule prevalence and characteristics in the general population. One of the potentially influencing factors on lung nodule presence in the general population is the presence of respiratory illnesses that depend on season. For that reason, we investigated if the season of year when respiratory illnesses are at their peak, are an influencing factor for the presence of LDCT-detected lung nodules in a general Dutch population. On the one hand, during the generally warmer summer months (April until the end of September), increased circulating pollen levels lead to an increase in the number of sufferers of allergic rhinitis (hay fever)^12^. On the other hand, in the Netherlands there is an annual influenza outbreak lasting on average 14 weeks (December to March), however cases have been reported as early as October^13,14^. Additionally, according to a recent study, the peak incidence of respiratory syncytial virus in the Netherlands is during the winter months (November until the end of February of the next year on average)^15^. Research has shown a greater number of hospitalisations due to community-acquired pneumonia during the winter than any other season^16^.

In this Imaging in Lifelines (ImaLife) sub-study, we sought to investigate whether presence and characteristics of LDCT-detected lung nodules varied by season in the general population.

## Results

### Population characteristics

Of the 3456 participants who had undergone a baseline low-dose CT chest scan between October 2018 and October 2019, 1497 (43%) were men, median age was 57 years (range from 45 to 88 years, inter-quartile range [IQR] 51–62), 611 (18%) were current smokers, 1346 (39%) were former smokers and 1464 (43%) had never smoked or had smoked for less than 1 year. Lung function test results showed a median FEV1 Forced Expiratory Volume in 1 s/FVC Forced Vital Capacity (FEV1/FVC) ratio of 76.8% (IQR 72.5–80.8%). The percentage of participants with FEV1/FVC ratio ≤ 70% (Global Initiative for Chronic Obstructive Lung Disease (GOLD) standard for confirmation of the presence of persistent airflow limitation^17^) was 17%. An overview of participant characteristics can be found in Table 1. Of all participants, 1312 (38%) had at least one lung nodule ≥ 30 mm^3^ (~ 3 mm in diameter); of these, 782 (60%) had one nodule, 266 (20%) had two nodules, 131 (10%) had three nodules, and 133 (10%) had four or more nodules ≥ 30 mm^3^. The median number of nodules per participant was one, and the range was from one to 21.

**Table 1:** Population characteristics.

Overall (n = 3456)		Season		Seasonal variation
		Summer (n = 1723)	Winter (n = 1733)	*p* value
**Baseline low-dose CT scan participants**				
Sex				
Female	1959 (57%)	985 (57%)	974 (56%)	0.804
Male	1497 (43%)	738 (43%)	759 (44%)	0.587
Age				
≤ 49	608 (18%)	254 (15%)	354 (20%)	< 0.001
50–54	737 (21%)	298 (17%)	439 (25%)	< 0.001
55–59	898 (26%)	366 (21%)	532 (31%)	< 0.001
60–64	559 (16%)	309 (18%)	250 (14%)	0.013
65–69	290 (8%)	226 (13%)	64 (4%)	< 0.001
≥ 70	364 (11%)	270 (16%)	94 (6%)	< 0.001
Median (IQR)	57 (51–62)	59 (53–66)	55 (50–59)	
Smoking status^a^				
Current	611 (18%)	274 (16%)	337 (20%)	0.011
Former	1346 (39%)	737 (43%)	609 (35%)	< 0.001
Never and smoked ˂ 1 year	1464 (43%)	696 (41%)	768 (44%)	0.060
Lung function (FEV1/FVC ratio (%))				
≤ 70%	592 (17%)	321 (18%)	271 (15%)	0.040
71–80%	1934 (56%)	967 (56%)	967 (56%)	1.000
81–90%	911 (26%)	427 (25%)	484 (28%)	0.059
> 90%	19 (1%)	8 (1%)	11 (1%)	0.491
Median (IQR)	76.8 (72.5–80.8)	76.6 (72.1–80.5)	77.2 (72.9–81.1)	

*N* total number, *IQR* inter quartile range, *FEV1* forced expiratory volume in 1 s, *FVC* forced vital capacity.

^a^Smoking status information missing for 35 participants.

### Seasonal influence on presence of lung nodules

Frequencies of LDCT scans performed, total nodules detected and participants with at least one lung nodule ≥ 30 mm^3^, per month and per season, can be found in the Table 2. Of the 3456 scans, 1723 (49.9%) scans were performed in summer (hay fever season) and 1733 (50.1%) were performed in winter (influenza season). Considerably fewer LDCT scans were performed in both August and October, when compared with other scan months, due to school holidays in these months.

**Table 2:** Distribution of baseline scans, total nodules detected and participants with ≥ 1 lung nodule > 30 mm^3^ (~ 3 mm in diameter).

Month/season	Total baseline scans performed (n = 3456)	Total nodules ≥ 30 mm^3^ detected (n = 2496)	Participants with ≥ 1 nodule ≥ 30 mm^3^ (n = 1312)
January	336 (10%)	206 (8%)	131 (10%)
February	257 (7%)	162 (6%)	84 (6%)
March	390 (11%)	295 (12%)	155 (12%)
April	313 (9%)	238 (10%)	126 (10%)
May	289 (8%)	249 (10%)	134 (10%)
June	380 (11%)	282 (11%)	153 (12%)
July	263 (8%)	229 (9%)	113 (9%)
August	98 (3%)	118 (5%)	49 (4%)
September	380 (11%)	281 (11%)	136 (10%)
October	83 (2%)	53 (2%)	28 (2%)
November	379 (11%)	192 (8%)	124 (9%)
December	288 (8%)	191 (8%)	79 (6%)
Summer	1723 (50%)	1397 (56%)	711 (54%)
Winter	1733 (50%)	1099 (44%)	601 (46%)
**Seasonal variation**			
*p* value	0.865	< 0.001	0.002
χ^2^	0.029	35.579	9.223

In total, 2496 lung nodules ≥ 30 mm^3^ were detected; 1397 (56%) in summer and 1099 (44%) in winter (*p* < 0.001). When looking at the number of participants with at least one lung nodule detected (n = 1312), 711 (54%) were recorded in the summer season which was significantly more than the 601 (46%) recorded in the winter season (*p* = 0.002).

**Figure 1:**
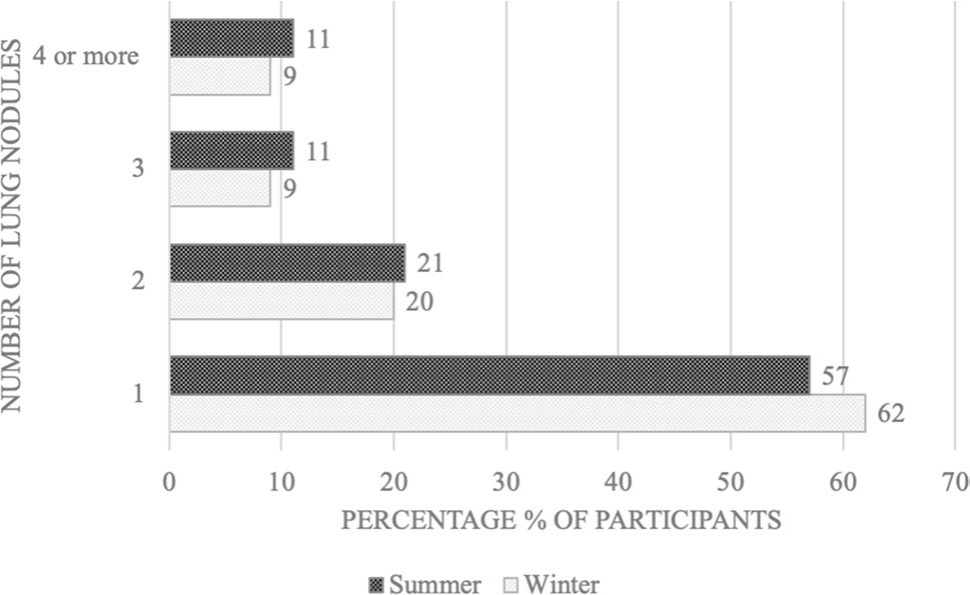
Percentage of participants per season with 1, 2, 3, or 4 or more nodules.

Moderate variation was observed in the seasonal distribution of specific number of nodules per participant (1, 2, 3, 4 or more) when compared to total participants with a nodule. More participants with one solitary nodule were recorded in winter (62%) compared to summer (57%), whereas more participants with either two, three, or four or more nodules were recorded in summer (21%, 11%, 11%) when compared to those recorded in winter (20%, 9%, 9%). An overview can be seen in Fig. 1.

In a subpopulation which may potentially be eligible for LDCT-lung cancer screening (n = 548), significantly more lung nodules ≥ 30 mm^3^ were detected in the summer (n = 251, 51%) than the winter (n = 245, 49%), (*p* = 0.04), similarly to the general population. The number of participants with at least one nodule ≥ 30 mm^3^, despite not being statistically significant, also showed a similar trend: higher in the summer months when compared to the winter months (117 vs. 108 participants respectively, *p* = 0.071).

### Seasonal influence on lung nodule characteristics

**Figure 2:**
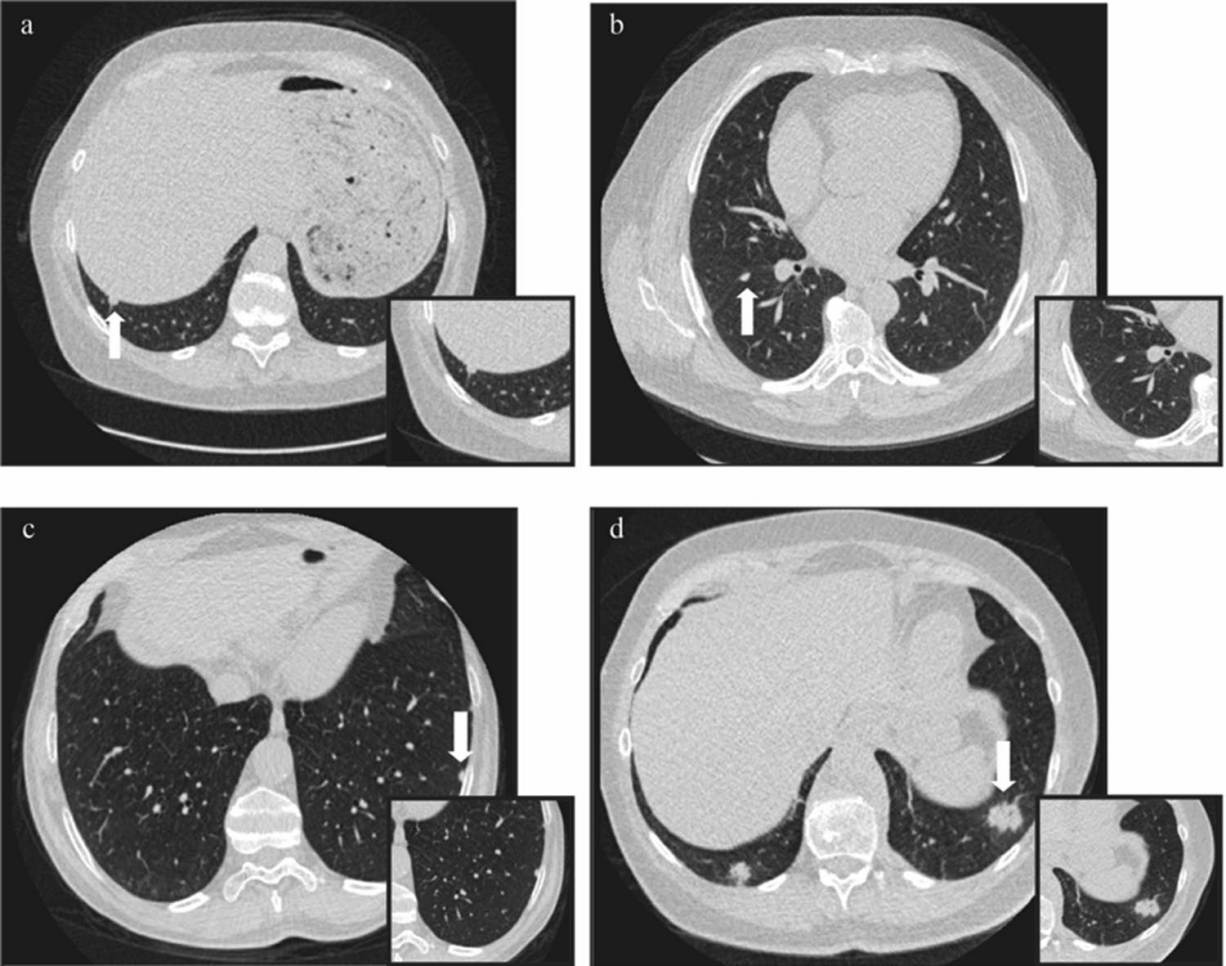
Transverse CT images of detected lung nodules in four ImaLife participants. Lung nodules indicated by white arrows, with zoomed-in view in the left bottom. (**a**) Solid, 136 mm^3^, oval, smooth edged, non-calcified, peripheral lung nodule, in the right lower lobe posterior basal segment. (**b**) Solid, 132 mm^3^, triangular, smooth edged, non-calcified, peripheral lung nodule which was categorised as a typical perifissural nodule (PFN), in the right lower lobe apical segment. (**c**) Solid, 154 mm^3^, triangular, smooth edged, non-calcified, peripheral lung nodule which was categorised as an atypical PFN, in the left lower lobe lateral basal segment. (**d**) Solid, 3933 mm^3^, irregular shaped, irregular edged, non-calcified peripheral nodule, situated in the left lower lobe anterior segment.

Lung nodule characteristics and seasonal distribution for the 2496 lung nodules detected can be found in Table 3. Overall, lung nodules detected were predominantly < 100 mm^3^ (~ 6 mm in diameter) (83%), solid (93%), smooth edged (87%), non-calcified (92%) and in the peripheral lung (83%). Furthermore, the majority of nodules had no features of a typical or atypical perifissural nodule (PFN) (64%). Example images of LDCT detected lung nodules from the ImaLife study can be seen in Fig. 2.

**Table 3:** Nodule characteristics and seasonal variation in size and characteristics.

Lung nodule characteristics		Season			
Overall (N = 2496)		Summer (N = 1397)	Winter (N = 1099)	χ^2^	*p* value
**Nodule size ((part) solid component)**	*n* = 2331	*n* = 1304	*n* = 1027		
< 100 mm^3^ (~ < 6 mm)	1922 (83%)	1081 (83%)	848 (83%)	0.001	0.972
100–299 mm^3^ (~ 6–8 mm)	344 (15%)	185 (14%)	159 (15%)	0.689	0.407
≥ 300 mm^3^ (~ > 8 mm)	54 (2%)	35 (3%)	20 (2%)	1.302	0.254
**Nodule type**					
Solid	2318 (93%)	1296 (93%)	1022 (93%)	0.008	0.931
Sub-solid (partial solid and pure ground glass)	169 (7%)	93 (7%)	76 (7%)	0.116	0.734
Partial solid	13 (< 1%)	8 (< 1%)	5 (< 1%)	0.162	0.687
Pure ground glass	156(6%)	85 (6%)	71 (6%)	0.145	*0.703*
**Nodule shape**					
Spherical	169 (7%)	68 (5%)	101 (9%)	**17.043**	**< 0.001**
Oval	663 (26%)	354 (25%)	309 (28%)	1.828	0.176
Triangular	963 (39%)	539 (39%)	424 (39%)	0.000	0.985
Polygonal	407 (16%)	248 (18%)	159 (14%)	**4.021**	**0.045**
Irregular	292 (12%)	187 (13%)	105 (10%)	**7.663**	**0.006**
**Nodule edge**					
Smooth	2179 (87%)	1205 (86%)	974 (89%)	0.433	0.511
Lobulated	49 (2%)	32 (2%)	17 (1%)	1.722	0.189
Spiculated	7 (< 1%)	3 (< 1%)	4 (< 1%)	0.491	0.484
Irregular	138 (6%)	82 (6%)	56 (5%)	0.655	0.418
Fuzzy	121 (5%)	74 (5%)	47 (4%)	1.306	0.253
**Lung segment**					
Right upper	516 (21%)	288 (21%)	228 (21%)	0.007	0.932
Right middle	336 (13%)	175 (13%)	161 (15%)	2.092	1.148
Right lower	566 (23%)	339 (24%)	227 (21%)	3.483	0.062
Left upper	444 (18%)	220 (16%)	224 (20%)	**7.498**	**0.006**
Left lower	624 (25%)	365 (26%)	259 (24%)	1.575	0.210
**Location within lung**					
Central	427 (17%)	235 (17%)	192 (18%)	0.161	0.688
Peripheral	2067 (83%)	1161 (83%)	906 (82%)	0.024	0.877
**Possible perifissural nodule (PFN)**					
No features of a PFN	1538 (64%)	954 (68%)	584 (53%)	**22.686**	**< 0.001**
PFN (both typical and atypical)	758 (30%)	383 (27%)	375 (34%)	**9.212**	**0.002**
Typical PFN	567 (22%)	307 (22%)	260 (24%)	0.792	0.373
Atypical PFN	191 (8%)	76 (5%)	115 (11%)	**20.367**	**< 0.001**
Not able to distinguish	118 (5%)	43 (3%)	75 (7%)	**9.212**	**0.002**
**Nodule calcification**					
None	2272 (92%)	1264 (91%)	1008 (92%)	0.124	0.725
Fully calcified	141 (6%)	78 (6%)	63 (6%)	0.027	0.871
Central	22 (1%)	18 (1%)	4 (< 1%)	**5.952**	**0.015**
Popcorn	1 (< 1%)	0	1 (< 1%)		
Rim	3 (< 1%)	2 (< 1%)	1 (< 1%)	0.139	0.71
Other	20 (< 1%)	17 (1%)	3 (< 1%)	**6.826**	**0.009**

*PFN* perifissural nodule; missing data (number cases): nodule size solid nodules (3), nodule type (6), nodule shape (2), nodule edge (2), lung segment (10), location within lung (2), possible pfn (82), calcification (37).

Significant seasonal variation in bold type.

Seasonal variation was observed in the shape of nodules and lung segment in which nodules were detected and in percentage of PFNs. When compared to the total lung nodules per season (summer n = 1397, winter n = 1099), more of the detected nodules were spherical in the winter months (9%) than in the summer months (5%) (*p* < 0.001), whereas relatively more polygonal and irregular nodules were present in summer (18% and 13%) than in winter (14 and 10%) (*p* = 0.045 and *p* = 0.006 respectively). There was a positive association between nodules detected in the left upper lobe and winter season (20%) when compared to the summer (16%) (*p* = 0.006), along with slightly more centrally calcified nodules (summer 1.3% vs. winter 0.4%, *p* = 0.015). Finally, when looking at perifissural nodules, relatively more non-PFN nodules were detected in the summer (68%) compared to the winter (53%) (*p* < 0.001), whereas relatively more atypical PFNs were present in winter (10%) than in summer (5%) (*p* < 0.001). Size, type, edge, and location (central vs. peripheral) of LDCT detected lung nodules did not significantly differ according to the season of year. Similar results were seen in the secondary analysis when looking at only the largest nodule per participant, with the exception of the lung segment and calcification of the nodules detected. See Supplementary Data S1.

**Figure 3:**
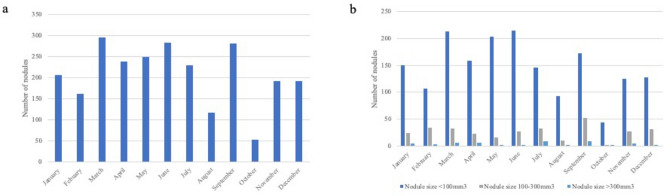
Monthly distribution of total nodules and non-calcified solid lung nodules detected in ImaLife participants based on nodule size categories. (**a**) Overview of the total number of lung nodules detected per month. (**b**) Overview of the number of lung nodules detected per nodule size category per month: nodule size < 100 mm^3^ (< ~ 6 mm), nodule size 100–300 mm^3^ (~ 6–8 mm) and nodule size > 300 mm^3^ (> ~ 8 mm).

When looking more specifically at lung nodule size categories, no statistically significant seasonal variation was seen. Overall, we can report for solid non-calcified nodules (n = 2107), 765 (44%) of the nodules < 100 mm^3^ (~ 6 mm) were detected in winter, compared to 988 (56%) in summer, (*p* = 0.761). Lung nodules which were 100–300 mm^3^ (~ 6–8 mm) were distributed quite evenly, with 149 (48%) detected in winter, compared to 159 (53%) detected in summer, (*p* = 0.122). In the category of nodules > 300 mm^3^ (~ 8 mm), 18 (39%) were detected in winter versus 28 (61%) detected in the summer. An overview of monthly distribution can be seen in Fig. 3.

### Multivariable binary regression analysis

In a multivariable binary logistic regression analysis, presence of at least one lung nodule in association with winter (influenza season), age, gender, smoking status, and FEV1/FVC ratio was studied, see Table 4. According to our analysis, season, gender, age and smoking status, after adjustment for cofounders, were all significant predictors of the presence of one or more LDCT detected lung nodules (*p* = 0.002, *p* < 0.001, *p* = 0.001, *p* = 0.011). Our results showed that after adjustment for gender, age, and smoking status, summer (hay fever) season significantly increased the likelihood of the presence of one or more lung nodules by roughly 20% (adjusted odds ratio [OR] 1.255, *p* = 0.002). Whereas female gender decreased the likelihood (OR 0.714, *p* < 0.001) after adjustment for season, age and smoking status. Increasing age (per 1-year increase in age OR 1.015, *p* = 0.001) and being a current or former smoker (OR 1.205, *p* = 0.011) significantly increased the likelihood of the presence of one or more LDCT detected lung nodules.

**Table 4:** Binary logistic multivariable regression analysis outcomes for the association between influenza (winter) season, participant characteristics and the detection of one or more lung nodules ≥ 30 mm^3^ (~ 3 mm in diameter).

Predictor	Sig	Exp(B)	95% C.I. for Exp(B)	
			Lower	Upper
Season (1 = summer, 0 = winter)	0.002	1.255	1.087	1.448
Age	0.001	1.015	1.006	1.024
Gender (1 = female, 0 = male)	< 0.001	0.714	0.620	0.821
Never smoker (1 = no, 0 = yes)	0.011	1.205	1.044	1.392

*Sig. p* value, *Exp(B)* odds ratio, *C.I. 95%* confidence interval.

## Discussion

The aim of this ImaLife sub-study was to explore whether the season of the year, related to incidence of respiratory illnesses, affects the prevalence of lung nodules found on low-dose CT chest scans in the general population. From our study we can report several interesting findings. Firstly, the prevalence of lung nodules detected was significantly greater in the summer (hay fever) season (56%) (April to September) than in the winter (influenza) season (44%). Secondly, summer season increased the likelihood of the presence of ≥ 1 lung nodule by approximately 20% (OR 1.255, *p* = 0.002), after adjustment for confounders. Thirdly, seasonal variation was seen in lung nodule characteristics, in particular shape, lobular location, calcification and PFNs.

The aforementioned findings on nodule presence indicate that allergic rhinitis (hay fever) could increase the prevalence of LDCT-detected lung nodules in the general population. Several articles have been published previously on the association between chronic rhinosinusitis and both CT pulmonary changes and lung cancer. One research article published in 2015, showed that CT pulmonary changes could be observed in patients with a diagnosis of chronic rhinosinusitis^18^. These pulmonary changes included: centrilobular nodules, atelectasis, ground-glass opacities, bronchial wall thickening, bronchiolectasis and air-trapping; they occurred more frequently in patients with a diagnosis of chronic rhinosinusitis than in those without. Furthermore, a study from 2008 in the Singapore Chinese population looked at the association between chronic rhinosinusitis (both allergic and non-allergic) and risk of lung cancer^19^. They found that chronic rhinosinusitis especially in woman may increase lung cancer risk. However, this study was not able to distinguish if the effect was from atopic conditions such as allergic rhinitis or from other conditions that cause chronic inflammation such as asthma^19^. A third study performed in 2016 in Taiwan also concluded that people who suffered from chronic rhinosinusitis are at a greater risk of developing lung cancer, specifically the subtype adenocarcinoma^20^. The authors of this study suggest that even though chronic rhinosinusitis is a disease-causing inflammation of the nose and sinuses, it may actually be representative of pan-airway inflammation. Chronic lower airway inflammation and lung cancer risk have been studied in more detail. Multiple studies on tuberculosis and chronic obstructive pulmonary disease patients have shown that especially in women, chronic lower airway inflammation can lead to an increased risk of lung cancer^21–24^. As upper and lower airways have similar pathophysiology^25^, it could be hypothesised that when an immunological response is triggered in the upper airways, there is an upregulation of the immunological system throughout the airways^20^. Should this process indeed occur, allergic rhinitis could be a trigger for lower airway inflammation. As the potential underpinning factor appears to be chronic inflammation, we could hypothesize that more CT-detected lung nodules or potentially lung cancers could be present in a population that are severe sufferers of allergic rhinitis. This population is more likely to be allergic for both tree and grass pollen, meaning their symptoms are present for a longer period of the year. A suggestion based on these results could be to further investigate a possible association between pollen count, allergic rhinitis and incidental lung nodule detection, and perhaps lung cancer.

With regards to the seasonal variation in lung nodule characteristics that we observed, characteristics associated with infection and inflammation were seen in both the hay fever (summer) and influenza (winter) season. Relatively more nodules of polygonal and irregular morphology were present in summer, when compared to winter. These lung nodules are shown to be possibly resolving and therefore could be related to infection^26^. On the other hand, relatively more perifissural nodules were detected in the winter season. These nodules are thought to be most likely related to intrapulmonary lymph nodes, which would suggest they are also associated with infection or inflammation^27^. As a potential explanation we can hypothesise that the underlying pathophysiology, be it a possible respiratory infection in winter or an allergic immunological response in the summer, could lead to varying lung nodule characteristics. Therefore, we are of the opinion that these findings provide a reason to perform short term follow-up LDCT, to look at the development of these nodules and their characteristics over time in order to elucidate their true underpinning pathophysiology.

This study was unique as the climate was the same for all participants and as this study investigates incidental lung nodules in the general population and not only a population at high-risk of developing lung cancer. However, our study did have limitations. Firstly, this study only looked at nodules greater than ≥ 30 mm^3^ (or about 3 mm diameter)^28^. This decision was made because it is likely that these nodules have the most clinical significance. Nevertheless, lung nodules below 30 mm^3^ will be detected in clinical practice and for that reason it may be of importance in future to also study these smaller nodules in more detail. The downside to this is that a high number of the general population are likely to have these very small lung nodules, which would make analyses extremely time consuming and costly, while it is not possible to accurately determine morphological details for these nodules. Additionally, at this current time there are not enough data available to study disappearance of CT-detected lung nodules in this population. It will be possible however to look at the resolution of nodules ≥ 100 mm^3^ (~ 6 mm in diameter) in this population in the future, as nodules of this size are followed up on short term using a repeat LDCT scan and volumetric nodule analysis.

We recommend that research continues to investigate any underlying causes for seasonal variation in CT-detected lung nodule presence and characteristics, and that this new information is used to develop a standardised LDCT detected lung nodule management strategy that can be used routinely in clinical practice. In addition to investigating possible causative factors of LDCT detected lung nodules, it would also be beneficial to know the outcomes of these nodules, for example if they resolved or were later diagnosed as lung cancer. This knowledge would help to further understand the progression of these nodules.

In conclusion, there are seasonal differences in presence and characteristics of CT-detected lung nodules in an asymptomatic general population. We detected significantly more pulmonary nodules on LDCT scans in the summer months when hay fever is considered to be most prevalent. Additionally, lung nodule characteristics including shape, location, calcification and perifissural nodules may also be influenced by season of year. We suggest that further research is performed using short-term follow-up LDCT with volumetric nodule analysis, to investigate the outcome and therefore underpinning pathophysiology that causes this trend.

## Methods

### Study and participants

We performed our study on ImaLife data generated from participants taking part in the Lifelines study. The Lifelines study is a large, multigenerational, longitudinal cohort study, which has recruited over 167,000 participants from the North of the Netherlands. Lifelines participant data include demographics, clinical biomarkers, environmental factors and lifestyle factors gathered at ongoing assessment rounds^29^. A total of 22,000 eligible Lifelines participants are invited to the ImaLife study. The ImaLife (Imaging in Lifelines) study design, population and recruitment strategies have been previously published^30^. In short, ImaLife is an on-going study, which began in 2017. In ImaLife, Lifelines participants are invited for a low-dose CT (LDCT) scan of heart and lungs, in order to obtain reference values for imaging biomarkers of lung nodules, emphysema and coronary calcium. The eligibility criteria are: Lifelines participants aged 45 years and above who completed a lung function test during the second-round assessment of the Lifelines study. Individuals who did not complete the lung function test (due to dizziness or hyperventilation), had a chest CT within the past year and pregnant women were excluded from taking part in the ImaLife study. A total of 12,000 participants are expected to take part in the on-going ImaLife study. The ImaLife study was approved by the University Medical Center Groningen medical ethics committee, and is registered with the Dutch Central Committee on Research Involving Human Subjects (https://www.toetsingonline.nl, Identifier: NL58592.042.16)^30^. All participants must give informed consent in order to participate in ImaLife.

**Figure 4:**
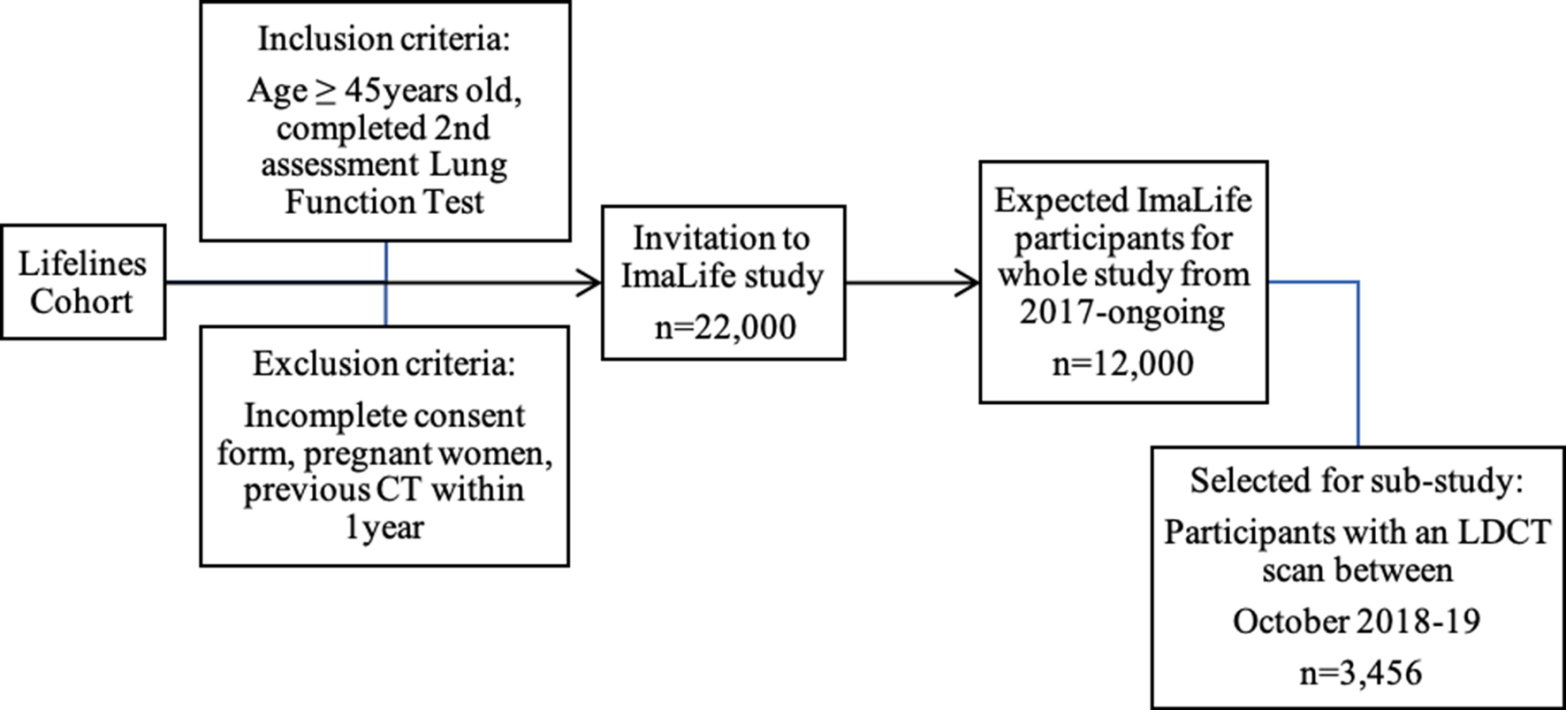
Overview of the ImaLife study and participants included in this sub-study. *LDCT* low-dose computed tomography.

In the current sub-study, we included 3456 asymptomatic ImaLife participants who underwent LDCT between October 2018 and October 2019 and had complete CT evaluation for lung nodules (see Fig. 4).

### Low-dose chest CT scan protocol

The image acquisition and analysis for the ImaLife study has been previously published^30^. ImaLife participants undergo a two-part low-dose CT using a third-generation dual- source CT scanner (SOMATOM Force, Siemens Healthineers, Germany). Firstly, participants undergo an electrocardiography (ECG) triggered non-contrast cardiac CT acquisition for coronary artery calcium scoring, followed by an LDCT chest acquisition. Scans are made at 120 kV, in high-pitch. Thoracic reconstructions are made with 1.0 mm slice thickness, 0.7 mm slice increment and both Br40 (medium smooth) and Qr59 (hard) reconstruction kernels^30^.

### Lung analysis and lung nodule characterisation

Radiologists with 6 to 12 years of experience and medical researchers under the supervision of radiologists and after adequate training, perform the lung analysis and nodule volumetric measurements for this study using Syngo.via software (version VB30) with MM Oncology application (Siemens Healthineers, Germany). For this study, one experienced radiologist with a minimum of 3-years post residency thoracic radiology experience performed the nodule annotations independently without the use of a computer aided detection system. Any uncertainties with regards to nodule characterisation or classification, or incidental findings were discussed with a second experienced radiologist. All nodules with a volume of at least 30 mm^3^ (approximately 3 mm diameter) are recorded and further evaluated for nodule characteristics. Characteristics which are reported include: size (volume and diameters), shape (spherical, oval, triangular, polygonal or irregular), edge (smooth, lobulated, spiculated, irregular or fuzzy), calcification pattern (none, fully calcified, popcorn, rim, or other calcification pattern), type (solid, part-solid or pure ground glass), location (lung segment, and central or peripheral) and perifissural nodule (PFN) classification (no features of a PFN, typical PFN, atypical PFN, or unable to distinguish). All lung nodule measurements and characteristics are recorded in the nodule management system.

The ImaLife study based its lung nodule size cut-offs on the categorization in the EU position statement on low-dose CT lung cancer screening^10^, namely below 100 mm^3^ (< ~ 6 mm in diameter), 100–300 mm^3^ (~ 6–8 mm in diameter), and above 300 mm^3^ (> ~ 8 mm in diameter). According to the ImaLife study protocol^30^, in case of a non-calcified solid nodule of 100–300 mm^3^, the participant is invited for a short-term repeat CT for research purposes (to study the persistence of nodules and nodule volume doubling time in the general population). In case of a noncalcified solid nodule > 300 mm^3^, the participant is recommended to visit the general practitioner and if indicated, to be referred to a pulmonologist.

### Data management

This study used information on lung nodule characteristics from the ImaLife data management system, which has been explained in detail previously^30^. Additionally, participant characteristics including age, gender, smoking status and lung function were stored and analysed using the Lifelines data management system. Validated data from a baseline smoking questionnaire was used for participant smoking status. Smoking status was defined as: having never smoked or smoked for less than 1-year, current smoker, or former smoker, and included the use of cigarettes, roll-up cigarettes, cigarillos, cigars or pipe tobacco.

### Seasonal influences

For this study, we were particularly interested in the influence of seasonal respiratory illnesses, in particular the influenza virus and hay fever (allergic rhinitis). Therefore, we used only two seasons (summer and winter). We defined the Influenza season (winter) as 1st October to 31st March, based on the Netherlands National Institute for Public Health and the Environment (RIVM) reported 2018–2019 Influenza period^13^, and the hay fever (summer) season as 1st April to 30th September, based on the yearly peak in pollen levels and subsequent increase in hay fever caases^12^. We analysed the number of participants with a nodule ≥ 30 mm^3^, number of nodules ≥ 30 mm^3^ and nodule characteristics per month and per season.

### Statistical analysis

The data analysis was performed using the statistical software SPSS version 26 (Statistical Package for Social Sciences) and a *p* value of < 0.05 was considered statistically significant. Absolute frequencies and percentages were reported using descriptive statistics. Analyses of seasonal variation in lung nodule presence (total nodules n = 2496, participants with at least one lung nodule ≥ 30 mm^3^ n = 1312) and the number of lung nodules detected per participant (categories 1, 2, 3, 4 or more) were performed using a Chi-square Goodness-of-fit test.

Analyses of lung nodule characteristics were performed on two levels, both of which included correction for unequal proportions of nodules detected per season during Chi-square Goodness-of-fit testing. The first analysis looked at seasonal variation in nodule characteristics in the total lung nodule population (n = 2496), and the second, see Supplementary Data S1, considered only the largest nodule detected per participant (n = 1312). The second analysis was performed to account for possible correlation of lung nodule characteristics in participants with multiple nodules.

To adjust for confounding population characteristics including: age, gender, smoking status and lung function on the seasonal prevalence of one or more lung nodule, a multivariable binary logistic regression analysis was also performed. In 35 (1%) of the 3456 participants who had undergone an LDCT chest scan, data for smoking was missing from the Lifelines database and subsequently these participants were removed prior to analysis.

Lastly, we performed a sub-analysis, on seasonal lung nodule presence, in participants which may potentially be eligible for LDCT-lung cancer screening (n = 548). We did not have information on all inclusion criteria available which are regularly used in lung cancer screening trial programmes, for example for ex-smokers we do not have the date of smoking cessation. For this sub-analysis we included participants aged 50–75 years, current, and former smokers with a 30-packyears smoking history independent of when they ceased smoking. As these participant characteristics have formed the basis of the inclusion criteria in several previous lung cancer screening trials, we hypothesise that this sub-group of participants could possibly be eligible for a lung cancer screening programme.

## Supplementary information

Supplementary Information.

## Data Availability

The data of this study are publicly available. For access to the data that support the findings of this study, the Lifelines research office can be contacted via www.lifelines.nl/researcher.
