# Supplementary material for: Seasonal prevalence and characteristics of low-dose CT detected lung nodules in a general Dutch population
Source: Sci Rep. 2021 Apr 28;11:9139. doi: 10.1038/s41598-021-88328-y (PMC8080793; doi:10.1038/s41598-021-88328-y)
Supplement: Supplementary file 1 — Supplementary Information. [file 41598_2021_88328_MOESM1_ESM.docx]

**Seasonal prevalence and characteristics of low-dose CT detected lung nodules in a general Dutch population.**

Harriet L. Lancaster^1^, Marjolein A. Heuvelmans^2^, Gert Jan Pelgrim^1^, Mieneke Rook^1,3^, Marius G.J. Kok^1^, Ahmed Aown^1^, Geertruida H. de Bock^2^, Maarten van den Berge^4^, Harry J.M. Groen^4^, Rozemarijn Vliegenthart^1^*

Affiliations

1 *﻿Department of Radiology, University of Groningen, University Medical Center Groningen, Groningen, The Netherlands*

2 *﻿Department of Epidemiology, University of Groningen, University Medical Center Groningen, Groningen, The Netherlands*

*﻿*3 *Martini Hospital Groningen, Groningen, The Netherlands*

*﻿*4 *Department of Pulmonary Diseases, University of Groningen, University Medical Center Groningen, Groningen, The Netherlands*

*Corresponding author

Prof. Dr. Rozemarijn Vliegenthart

*Department of Radiology, University of Groningen, University Medical Center Groningen, Hanzeplein 1, 9713 GZ Groningen, The Netherlands*

﻿*r.vliegenthart@umcg.nl*

**Supplementary Data Analyses S1.**

***Analyses of seasonal influence on lung nodule characteristics after selecting only the largest lung nodule per participant.***

*S1.1 Methods*

From the total lung nodules detected (n=2464), participants with multiple nodules (n=530) were identified and the largest of those nodules was selected for further analyses in combination with participants with only one nodule (n=782). A total of 1312 lung nodules were included for the subsequent analysis using a Chi-square Goodness-of-fit test.

*S1.2 Results*

Seasonal variation was observed in the shape of nodules, and PFNs. When compared to the number of nodules (largest only) per season (hay fever season (summer) n=711, influenza season (winter) n=601), relatively more of the spherical nodules detected were present in the winter months (9%) than in the summer months (5%), *p=0.004*, whereas relatively more of the polygonal nodules were present in the summer (20%) than the winter (15%), *p=0.03*). There was also a positive association between nodules with no features of PFNs and the summer season (66%) compared to the winter (54%), *p=0.003*, whereas relatively more of the atypical PFNs were present in the winter (10%) than the summer (6%), *p=0.007*.

Size, type, edge, calcification and location (both segmental and central vs peripheral) of LDCT detected lung nodules were not significantly associated with the season of year.
